# Evaluation of the antibacterial activity of *Lactobacilli* probiotics supernatants against *Enterococcus faecalis* (in-vitro study)

**DOI:** 10.1186/s12903-022-02434-5

**Published:** 2022-09-18

**Authors:** Shymaa Shaaban, Gamal M. Hamad, Salma Genena, Marwa A. Meheissen, Sybel Moussa

**Affiliations:** 1 Division of Endodontics, Conservative Dentistry Department, Faculty of Dentistry, Alexandria University, Azarita, Alexandria, 21527 Egypt; 2 Department of Food Technology, Arid Lands Cultivation Research Institute (ALCRI), City of Scientific Research and Technological Applications (SRTACity), New Borg El-Arab, Alexandria, 21934 Egypt; 3 Medical Microbiology and Immunology Department, Faculty of Medicine, Alexandria University, Alexandria, 21512 Egypt

**Keywords:** Calcium hydroxide, Endodontics, *Enterococcus faecalis*, *Lactobacilli*, Probiotics

## Abstract

**Background:**

There is an increasing demand to find a potent antibacterial agent against endodontic pathogens with the least toxic effect. The study aimed to evaluate the antibacterial activity of *Lactobacilli* probiotics on *Enterococcus faecalis* (*E. faecalis)* in comparison to calcium hydroxide paste.

**Methods:**

The study involved two stages; (stage one): determination of the antibacterial properties of three strains of *Lactobacilli* probiotics supernatants (PS); *Lactobacillus plantarum*, *Lactobacillus rhamnosus*, *Lactobacillus acidophilus,* and a cocktail mix of the three supernatants against *E. faecalis* using agar well diffusion method in both solution and gel phase. The formed zones of inhibition (ZOI) were measured in millimeters (mm) and compared to each other. PS solution and gel of the largest ZOI were further compared with calcium hydroxide paste (35% Ultra Cal XS Ca(OH)_2_) This was followed by (stage two): assessment of the minimum inhibitory concentration (MIC) of the PS that showed the largest ZOI against *E. faecalis* by agar well diffusion assay for both PS solution and gel.

**Results:**

All supernatants showed growth inhibition against *E. faecalis,* and the cocktail mix showed the largest ZOI. However, no significant difference was found between the supernatants in both the solution and gel phases (*p* > 0.05). Ca(OH)_2_ showed a significantly lower effect than both the cocktail mix solution and gel (*p* < 0.05). The MIC of the cocktail mix solution and gel against *E. faecalis* was 50 mg/ml. (*p* > 0.05).

**Conclusion:**

PS has an antibacterial effect on *E. faecalis* and was more effective than Ca(OH)_2._ *Lactobacilli* probiotics could be a promising antibacterial agent used as an irrigant or an intracanal medication.

## Background

Failure of endodontic therapy is attributed to the survival of microorganisms in the root-filled teeth [1–3]. Removing all the intra-radicular bacteria is impossible because of the complex anatomy of the root canal system, including accessory canals, isthmus, and apical ramification, which almost limit the effect of mechanical preparation and chemicals used in the main canal [4, 5].

*Enterococcus faecalis* (*E. faecalis*) is one of the most commonly isolated bacteria from failed root canal treatments because of its virulence factors [1, 6, 7]. *E. faecalis* is a gram-positive facultative anaerobe with high survival mechanisms [6–8]. It forms a resistant biofilm that deeply invades the dentinal tubules and can adapt to extreme environmental conditions withstanding long periods of low nutrition [8, 9]. The presence of a proton pump is considered the primary resistance mechanism of *E. faecalis* to many intra-canal medicaments and irrigants [9, 10].

Calcium hydroxide (Ca(OH)_2_) is the most widely used intracanal medication in endodontics owing to its antimicrobial effect [11]. Its high alkalinity produced by the release of hydroxyl ions is responsible for destroying the bacterial cell membrane, DNA, and protein structure [11–13]. Despite its advantages, *E. faecalis* is resistant to the high pH of Ca(OH)_2_ [9, 13].

Probiotics are living microorganisms, mainly bacteria, that provide beneficial health effects to individuals and are widely used in dietary products [14, 15]. The World Health Organization (WHO) defined probiotics as “live microorganisms which, when administered in adequate amounts in food or as dietary supplement confer a health benefit on the host” [16]. The most commonly used strains of probiotics are lactobacilli (lactic acid) bacteria and bifidobacteria [15, 17].

The era of biological medications is not new since live microorganisms (probiotics) were successfully used in controlling gastrointestinal diseases, including infantile diarrhea, necrotizing enterocolitis, antibiotic-associated diarrhea, Rotavirus diarrhea, *Helicobacter pylori* infections as well as traveler's diarrhea [18, 19]. They have been used to inhibit *Clostridium perfringens* poultry meat infection and control food contamination and aflatoxin production [20, 21].

Probiotics have been previously used for treatment of oral health diseases [22]. They were used in caries control management [23, 24], treatment of halitosis, and oral candidiasis [25, 26] and have shown reasonable results when used as an adjunctive treatment for periodontitis [27–29]. However, their application in endodontic treatment still needs validation [30]. Hammad’s study in 2013 did not show an inhibitory effect of probiotics against *E. faecalis* [31], while other recent studies stated that probiotics were effective against endodontic pathogens [32–37]. Supplementation with probiotics significantly reduced inflammation and bone resorption in rats with apical periodontitis, suggesting their possible role in reducing the severity of apical periodontitis [38, 39]. Consequently, probiotics can be used as irrigants, intracanal medications, or even in regenerative endodontics because of their antibacterial and anti-inflammatory properties [15, 32, 35].

*Lactobacillus* strains are the most typical species of probiotics as they are desirable members of the intestinal microflora and have been “Generally Recognized As Safe” (GRAS) status [20, 40]. The strong antagonistic effects of *Lactobacillus* against a wide range of human pathogens make it a potential regimen for treatment and prevention of infections [40, 41].

With the limitation of Ca(OH)_2_ use against *E. faecalis* and the demand for a new medication to eradicate all bacteria inside the root canal system; the study aimed to assess and compare the antibacterial effect of *Lactobacilli* probiotics medication and Ca(OH)_2_ paste against *E. faecalis*.

The null hypothesis of the current study was that there would be no difference in the antibacterial effect of *Lactobacilli* probiotics in solution and gel forms against *E. faecalis* compared to Ca(OH)_2_ paste.

## Methods

### Study design

**Fig. 1:**
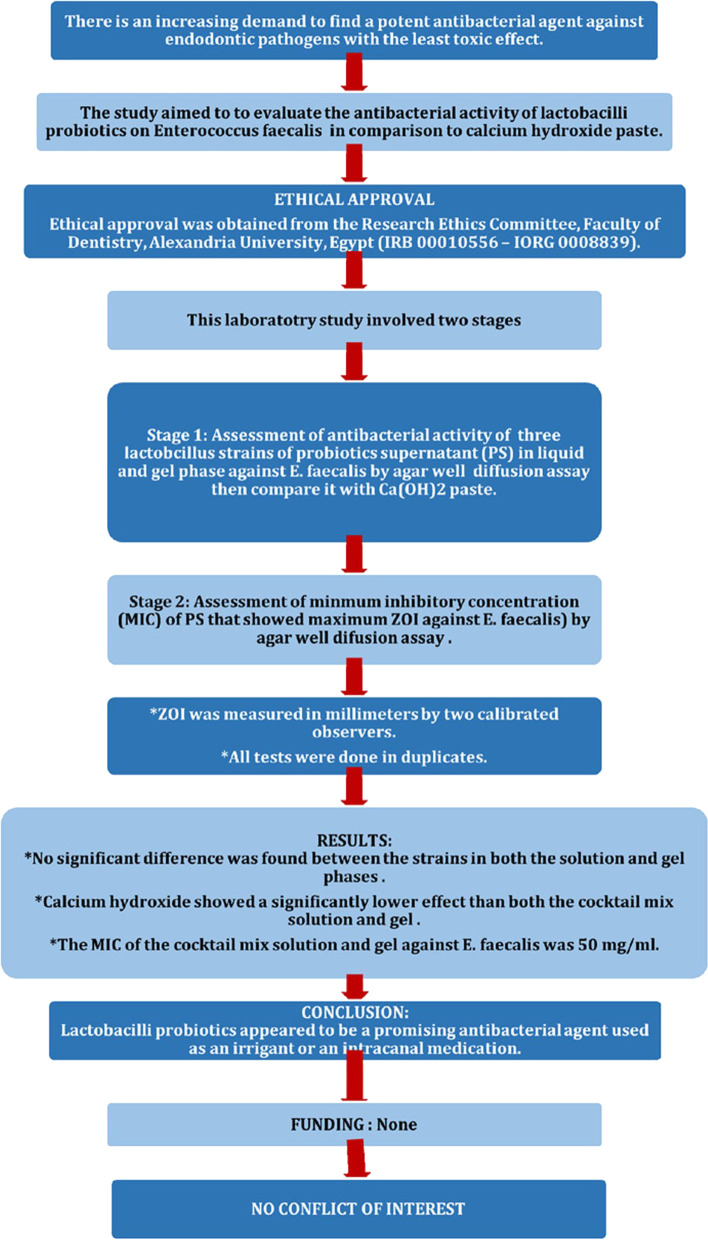
Study design according to PRILE guidelines

The manuscript of this laboratory study has been written according to Preferred Reporting Items for Laboratory studies in Endodontology (PRILE) 2021 guidelines [42]. (Fig. 1).

All tests were done in duplicates, and data were collected by two calibrated observers and presented as mean and standard deviation.

### Probiotics culture and supernatant preparation

Three strains of *Lactobacilli* Probiotics (*Lactobacillus plantarum* ATCC 14,917, *Lactobacillus rhamnosus* ATCC 7469, and *Lactobacillus acidophilus* ATCC 4356) were obtained from Microbiological Resources Centre (MIRCEN), Faculty of Agriculture, Ain Shams University, Cairo, Egypt. They were grown individually on De Man, Rogosa, and Sharpe (MRS) broth (HiMedia Laboratories Pvt. Ltd, India) aerobically for 48 h at 37 °C. The concentration of bacteria was OD_600_ = 0.6 for each strain. The pure isolate of Lactobacilli species suspension was propagated in a 100 ml flask containing MRS broth and incubated for 72 h at 37 °C. The cell-free supernatant (CFS) was obtained by centrifuging the culture at 10,000 rpm for 10 min and sterilized by syringe filter size 0.45 micron,, then lyophilized using a vacuum freeze dryer (Model FDF 0350, Korea) [20, 21].

### Poloxamer gel preparation

Poloxamer 407 powder was purchased from Sigma Aldrich Chemical Co., Gillingham, UK. Poloxamer gel was prepared using the 'cold technique’ by adding poloxamer 407 powder gradually to cold water (4–8 °C) under magnetic stirring up to a final concentration of 30% (w/w) poloxamer and kept in the refrigerator overnight to be ready for use [43–45].

This study was conducted on two stages under a sterile aseptic condition in a biosafety cabinet (Class II, Thermo scientific microbiological safety cabinet, Germany):

#### Stage 1: assessment of antibacterial activity of probiotics supernatants (PS) in liquid and gel phase against *E. faecalis* then compare it with Ca(OH)_2_ paste

##### PS solution

The lyophilized CFS of each of the three supernatants was mixed individually with sterile water to obtain PS solution of 200 mg/ml concentration. Also, a cocktail mix of the three supernatants was further obtained by combining equal volume of each CFS (200 mg/ml). The antibacterial activity of the three prepared PS, and the cocktail mix (*Lactobacillus plantarum* (LP), *Lactobacillus rhamnosus* (LR) and *Lactobacillus acidophilus* (LA)) were tested for their antibacterial effect against *E. faecalis* using agar well diffusion method [20, 33].

*E. faecalis* (ATCC 29,212) (American Type Culture Collection, Manassas, Virginia, USA.) was chosen for this study as an example of an endodontic pathogen and grown on a blood agar (HiMedia Laboratories Pvt. Ltd, India) plate at 37 °C for 24 h. A bacterial suspension of *E. faecalis* was prepared and adjusted to half MacFarland standard (1.5 × 10^8^ CFU/ml) using DEN-1, MacFarland densitometer (biosan, SIA, Latvia).

An agar plate was seeded with the prepared bacterial culture suspension of *E. faecalis* using a sterile cotton swab. Five wells were made on the agar plate using a cork borer of size 6 mm, four experimental wells and one control. The supernatants were loaded to each separate well (200 μl) as follows; 1: LP, 2:LR, 3: LA, 4: cocktail mix and, 5: sterile water (control) then incubated at 37 °C /24 h in an incubator (BINDER, Tuttlingen, Germany) [21].

##### PS gel

The lyophilized CFS of each of the three supernatants was mixed individually with poloxamer gel to get PS gel at 200 mg/ml concentration. A cocktail mix gel of the three supernatants was obtained at the same concentration.

The antibacterial activity of PS gel was also tested against *E. faecalis* in another agar plate using agar well diffusion assay as previously mentioned, four experimental wells and one control. The wells were loaded as follows; 1: LP (PS) gel, 2:LR (PS) gel, 3: LA (PS) gel, 4: cocktail mix gel and, 5: poloxamer gel (control) and incubated at 37 °C /24 h in an incubator (BINDER, Tuttlingen, Germany) [21].

For both plates, the formed zone of inhibition (ZOI) was recorded by two calibrated observers, measured in millimeters (mm), and compared to evaluate the antibacterial potential of each supernatant. The largest ZOI would indicate the most efficient PS in both gel and solution phases that would be further tested for the minimum inhibitory concentration (MIC) [21, 33].

##### Comparison of the antibacterial activity of PS solution, gel, and Ca(OH)_2_ paste against *E. faecalis*

The PS that showed the largest ZOI in solution and gel forms were compared to Ca(OH)_2_ paste (Ultra Cal XS Ca(OH)_2_) **(**Ultra dent Products Inc., South Jordan, UT, USA.).

An agar plate was seeded with a bacterial culture of *E. faecalis* using a sterile cotton swab. Three wells were made on the agar plate by using a cork borer of size 6 mm, one well was loaded with PS solution, the second was loaded with PS gel, and the last was loaded with Ca(OH)_2_ paste (35% Ultra Cal XS Ca(OH)_2_)**.**

The plate was then incubated at 37 °C/24 h in an incubator (BINDER, Tuttlingen, Germany). The formed zones were recorded and measured in millimeters by two calibrated observers. The largest ZOI indicated the most potent antibacterial agent against *E. faecalis*.

#### Stage 2: assessment of MIC of PS that showed maximum ZOI against E. faecalis

##### MIC of PS solution

The PS that showed the maximum antibacterial activity against *E. faecalis* was further assessed for its MIC using descending concentrations from 200 to 3.10 mg/ml diluted using sterile water. The prepared concentrations were tested for their antibacterial activity against *E. faecalis* using agar well diffusion assay as mentioned above. Two calibrated observers recorded the ZOI and determined the MIC as the least concentration of PS that showed ZOI [20, 21].

##### MIC of PS gel

The MIC of PS gel that displayed the largest ZOI against *E. faecalis* was determined using descending concentrations from 200 to 3.10 mg/ml. The anticipated descending concentrations were obtained by adding poloxamer powder 30% to the previously prepared different concentrations of PS solution and kept in the refrigerator overnight. Consequently, the well diffusion assay was performed, and the formed zones were recorded. MIC was obtained and compared with the MIC of PS solution [20, 21].

##### Assessment of the MIC of PS against *E. faecalis* by broth microdilution method

The MIC of PS for *E. faecalis* was also tested using the broth microdilution (BMD) method according to CLSI (Clinical Laboratory Standards Institute) guidelines [46]. Two-fold serial dilutions ranging from 200 to 0.10 mg/ml for PS were performed using cation-adjusted Mueller–Hinton broth (CAMHB). The *E. faecalis* inoculum was prepared to give a final concentration of 5 × 10^5^ CFU/ml in microtiter plate wells. The results were read for turbidity, MIC was determined as the least concentration of PS cocktail that inhibited visible growth of the organism and compared with the MIC obtained by agar well diffusion assay. In order to define the minimum bactericidal concentration (MBC) activities of the tested PS, the content of the clear wells was thoroughly mixed, and 10 μl were inoculated onto blood agar, incubated at 37 °C for 24 h and examined for 99.9% kill on the next day.

### Statistical analysis

Data were analyzed using IBM SPSS for Windows (Version 23.0). Normality was checked for all variables using descriptive statistics, plots (Q-Q plots and histogram), and normality tests. Means and standard deviation (SD) were calculated for all variables. Comparisons of ZOI of different PS supernatants and MIC concentrations in solution and gel were done using the independent samples t-test, while the comparison of ZOI between different supernatants in both solution and gel groups was done using one-way ANOVA test. Comparison of ZOI between Ca(OH)_2_, PS gel and PS solution groups, and comparisons of MIC of different concentrations in solution and gel groups were done using one-way ANOVA, followed by multiple pairwise comparisons using Bonferroni adjusted significance levels (for significant results). Significance was set at *p* value < 0.05.

## Results

### Stage 1. antibacterial activity of PS against E. faecalis

**Fig. 2:**
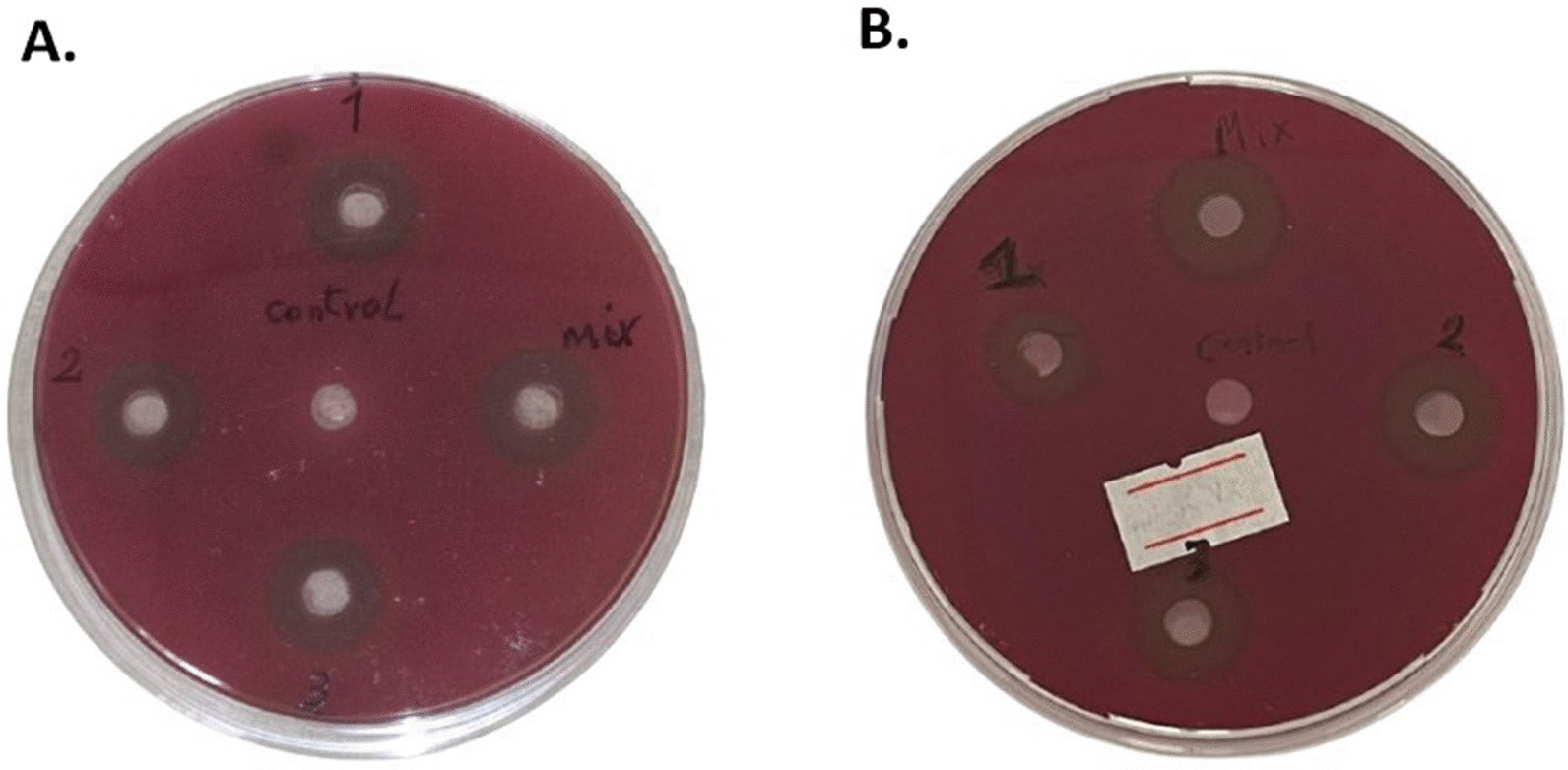
Antibacterial effect of different PS supernatants in solution and gel forms against *E. faecalis****.* A**: ZOI of the antibacterial activity of four experimental groups of PS solution. 1: *Lactobacillus plantarum*, 2: *Lactobacillus rhamnosus*, 3: *Lactobacillus acidophilus*, 4 (Mix): Cocktail mix and one control group: distal water. The cocktail mix showed the largest ZOI (16 mm). **B**: ZOI of the antibacterial activity of four experimental groups of PS gel. 1: *Lactobacillus plantarum*, 2: *Lactobacillus rhamnosus*, 3: *Lactobacillus acidophilus*, 4 (Mix): Cocktail mix and one control group: poloxamer gel. The cocktail mix showed the largest ZOI (15 mm). *The label was set to mark the plate with PS gel after the calibrated observers measured the ZOI

Figure 2 displayed the antibacterial effect of the three PS and the cocktail mix of the three supernatants (*Lactobacillus plantarum*; LP ATCC 14,917, *Lactobacillus rhamnosus*; LR ATCC 7469 and *Lactobacillus acidophilus;* LA ATCC 4356) in solution and gel phases by using agar well diffusion assay. It was observed that all supernatants and cocktail mixes showed growth inhibition of *E. faecalis* with obvious ZOI in both solution and gel forms.

It is noteworthy to mention that the cocktail mix of the three supernatants (LP, LR, LA) had the highest antibacterial activity as it showed the largest ZOI against *E. faecalis* in both solution and gel phases, thus was selected for further assessment tests.

Table 1 showed a non-significant difference between each of the three supernatants/cocktail mixes, neither in solution nor in gel phases (p > 0.05).

**Table 1:** Comparison of ZOI (in millimeters) of PS strains against *E. faecalis*

Supernatant	Solution	Gel	T- test *p* value
	(Mean ± SD)		
*Lactobacillus plantarum* (1)	14.00 ± 0.00	13.00 ± 0.00	0.24
*Lactobacillus rhamnosus* (2)	14.00 ± 1.41	13.50 ± 0.71	0.50
*Lactobacillus acidophilus* (3)	13.50 ± 0.71	12.00 ± 0.00	0.36
Cocktail mix	14.83 ± 1.60	14.00 ± 1.55	0.43
Control	ND	ND	–
One-way ANOVA *p* value	0.34	0.26	

Data represented are the means of duplicates ± standard error of means

*ND* Not detected

#### *Antibacterial potentials of PS solution, PS gel and Ca(OH)*_*2*_* against E. faecalis*

**Fig. 3:**
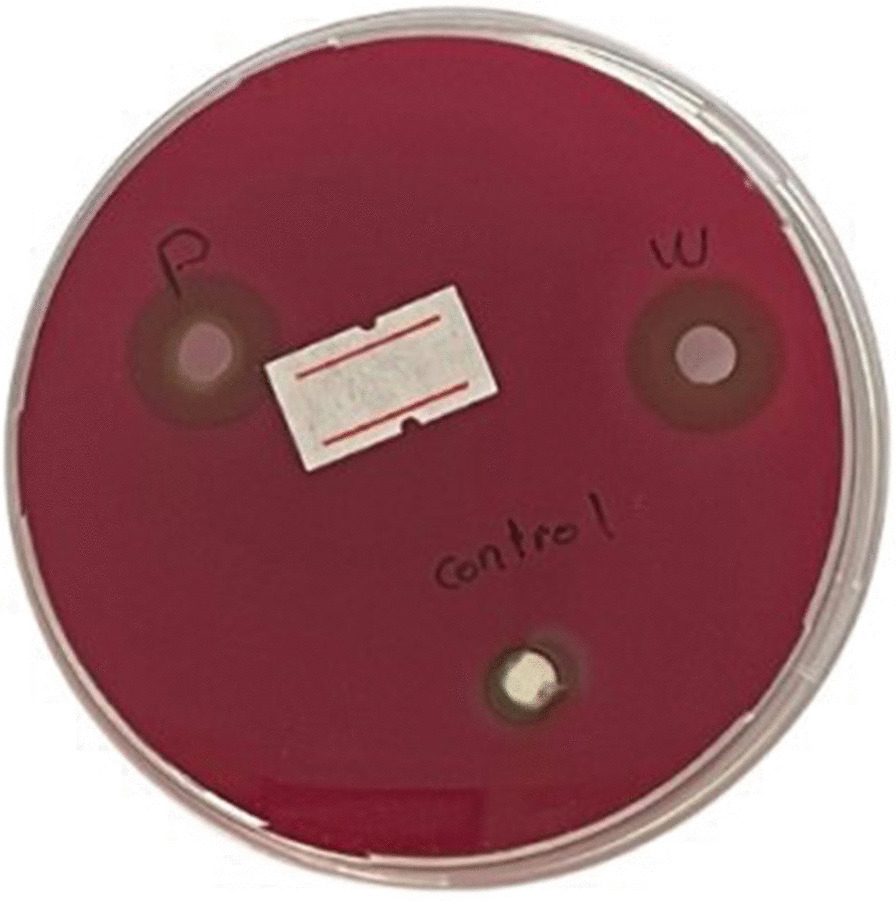
Antibacterial potentials of PS solution, PS gel and Ca(OH)_2_ paste against *E. faecalis*. ZOI of the antibacterial activity of three experimental groups: W:(PS) solution, P:(PS) gel and control: Ca(OH)_2_ paste, the calcium hydroxide showed the least ZOI (10 mm). * The label was set to mark the plate with PS gel after the calibrated observers measured the ZOI

The antibacterial activity of the two experimental groups (PS cocktail solution, PS cocktail gel) and one control: Ca(OH)_2_ paste was assessed by agar well diffusion assay as shown in Fig. 3.

The largest ZOI indicated the most potent antibacterial effect against *E. faecalis*, Ca(OH)_2_ paste was found to be the least potent agent.

There was a significant difference in the antibacterial activity between PS solution, PS gel and calcium hydroxide (35% Ultra Cal XS Ca(OH)_2_ (*p* < 0.05), however, there was no significant difference in the antibacterial activity between PS solution and PS gel (*p* > 0.05) as shown in Table 2.

**Table 2:** Comparison of ZOI (in millimeters) of PS cocktail solution, PS cocktail gel and Ca(OH)_2_ paste against *E. faecalis*

	PS solution	PS gel	Ca(OH)_2_ paste	One Way ANOVA *P* value
Mean ± SD	14.83 ± 1.60 b	14.00 ± 1.55 b	10.50 ± 0.71 a	0.02*

Data represented are the means of duplicates ± standard error of means

a, b Different letters denote statistically significant differences between groups using Bonferroni adjusted significance levels

*Statistically significant at *p* value < 0.05

### Stage 2. the MIC of the PS against E. faecalis by agar well diffusion assay

**Fig. 4:**
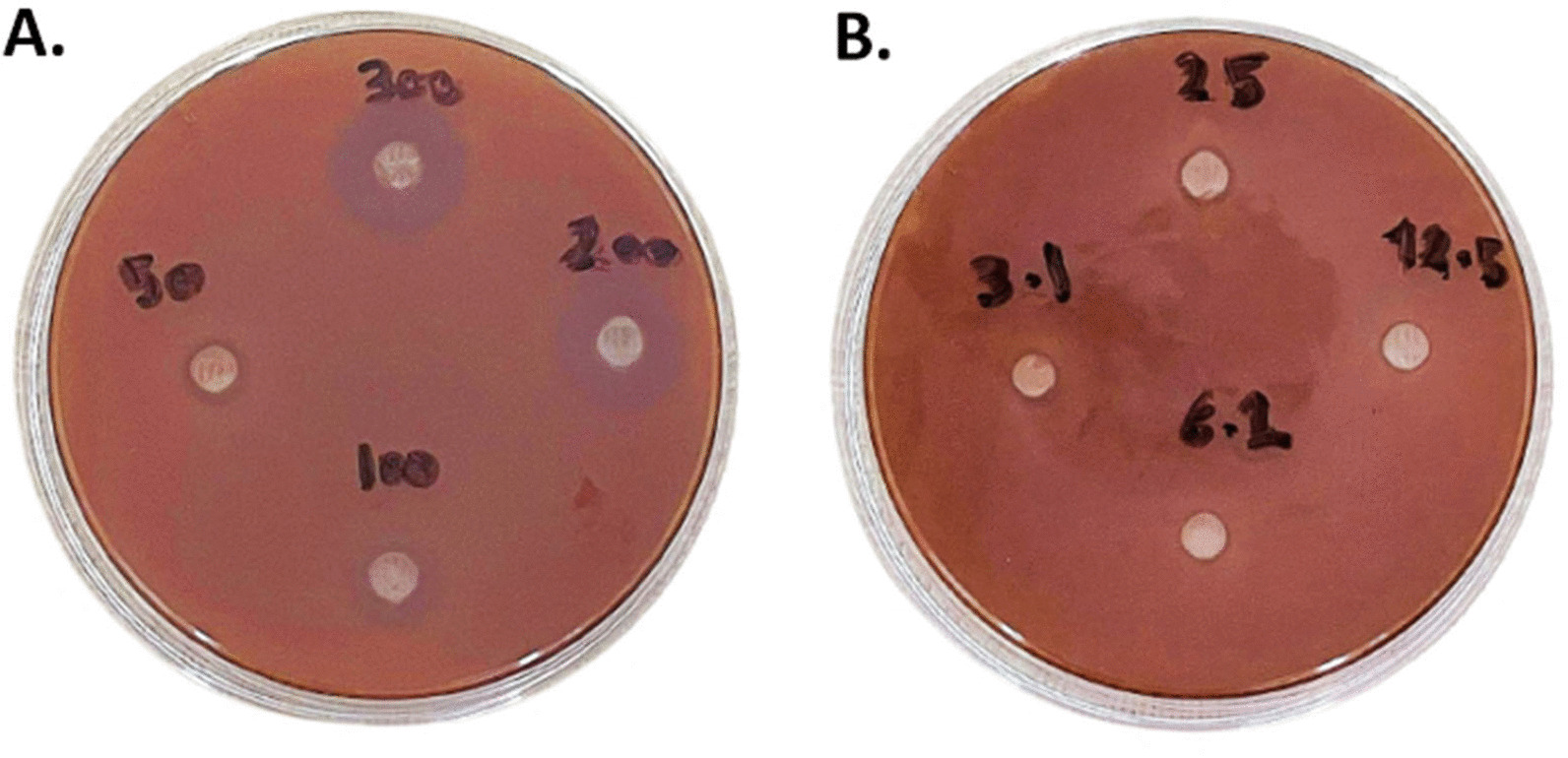
The MIC of PS cocktail solution against *E. faecalis* by agar well diffusion assay. **A** Showed ZOI of PS solution descending concentration 300 mg/ml, 200 mg/ml, 100 mg/ml, and 50 mg/ml. MIC was 50 mg/ml with 8 mm ZOI. **B** Showed no ZOI of concentrations 25 mg/ml, 12.5 mg/ml, 6.2 mg/ml, and 3.1 mg/ml

**Fig. 5:**
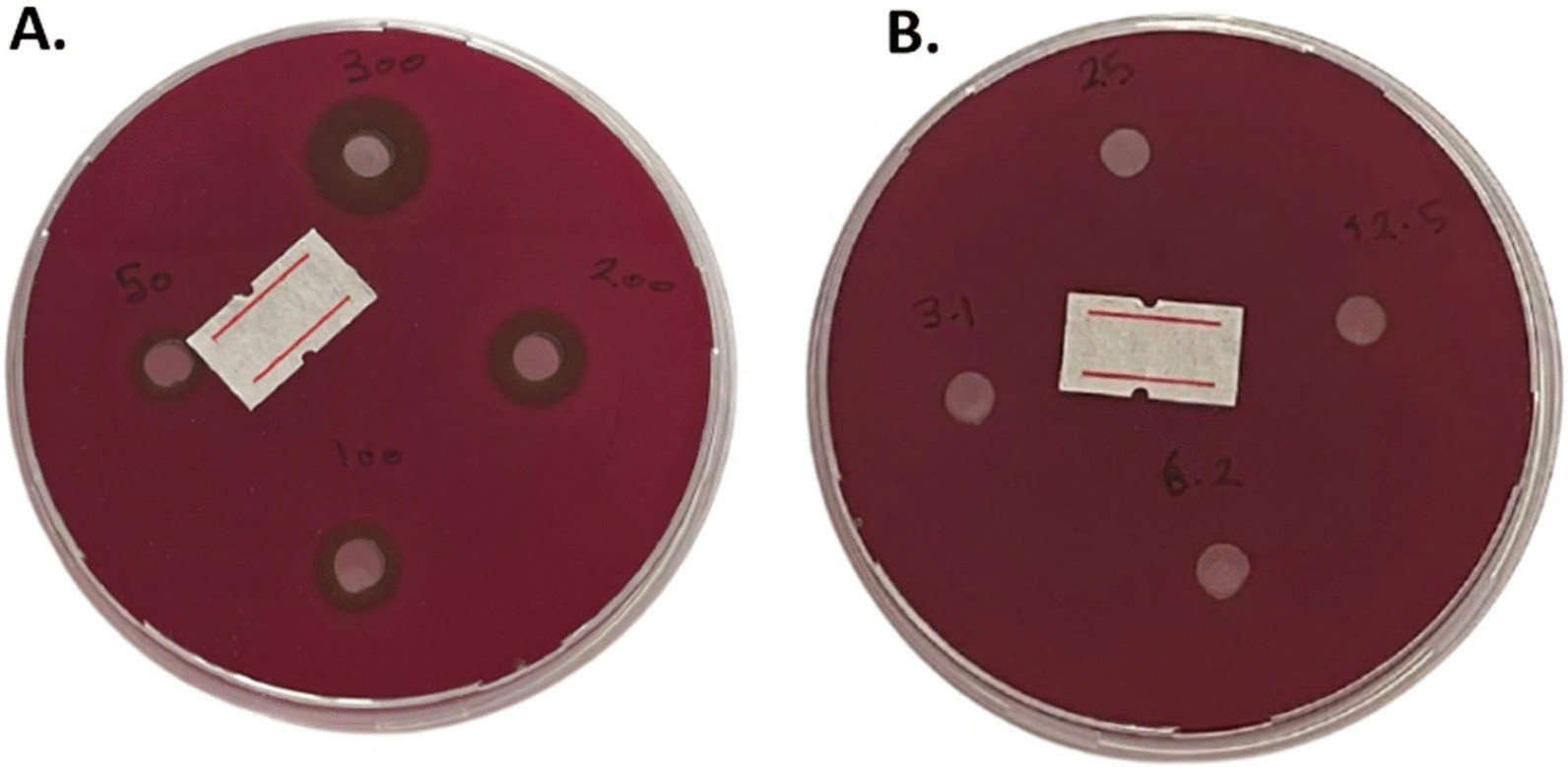
The MIC of PS cocktail gel against *E. faecalis* by agar well diffusion assay. **A** Showed ZOI of PS gel descending concentration 300 mg/ml, 200 mg/ml, 100 mg/ml, and 50 mg/ml. MIC was 50 mg/ml with 7 mm ZOI. **B** Showed no ZOI of concentrations 25 mg/ml, 12.5 mg/ml, 6.2 mg/ml, and 3.1 mg/ml. *The label was set to mark the plate with PS gel after the calibrated observers measured the ZOI

The MIC of PS cocktail mix (LP, LR, LA) denoting antibacterial activity was determined by using descending gradient concentrations from 200 to 3.10 mg/ml of the cocktail mix for the solution and gel forms. The MIC was (50 mg/ml) for both gel and solution forms without any significant difference between them (*p* > 0.05). (Figs. 4 and 5).

Table 3 revealed a significant difference between 300, 100 and 50 mg/ml concentrations, but no difference existed between 300 and 200 mg/ml in both solution and gel forms.

**Table 3:** MIC of PS cocktail solution and gel against E. faecalis

Concentration mg/ml	PS solution	PS gel	T-test *p* value
	ZOI (in millimeters) Mean ± SD		
300	15.50 ± 0.71 a	14.50 ± 0.71 a	0.10
200	14.83 ± 1.60 a	14.00 ± 1.55 a	0.11
100	10.50 ± 0.71 b	8.50 ± 0.71 b	0.38
50	8.50 ± 0.71 b	7.00 ± 0.00 b	0.29
25	ND	ND	–
12.5	ND	ND	–
6.5	ND	ND	–
3.1	ND	ND	–
One-Way ANOVA	0.001*	< 0.001*	

Data represented are the means of duplicates ± standard error of means

*ND* Not detected

^*^Statistically significant at *p* value < 0.05

a,b: Different letters denote statistically significant differences between different concentrations in each group using Bonferroni adjusted significance levels

#### The MIC of PS cocktail against E. faecalis by broth microdilution method

In Table 4, The MIC of the PS cocktail solution only was determined (50 mg/ml) and was similar to those obtained by agar diffusion assay. The result of the MBC confirmed the result of MIC.

**Table 4:** The MIC of PS cocktail against *E. faecalis* by agar well diffusion assay and broth microdilution method

Agar well diffusion assay method		Broth microdilution method	
Concentration (mg/ml)	ZOI (Y/N)	Concentration (mg/ml)	Visible growth (Y/N)
300	Y	300	N
200	Y	200	N
100	Y	100	N
50 (MIC)	Y	50 (MIC)	N
25	N	25	Y
12.50	N	12.50	Y
6.25	N	6.25	Y
3.13	N	3.13	Y
		1.50	Y
		.80	Y
		.40	Y
		.20	Y
		0.10	Y

^*^Y: Yes, N: No, MIC was the same for both tests

## Discussion

Since persistent bacteria in the root canal system is the main cause of root canal failure, there is an increasing demand to find a potent antibacterial agent against most endodontic pathogens with less toxic effects [1, 2]. Subsequently, this study was conducted to assess the antibacterial effect of *Lactobacilli* probiotics as a potential endodontic medication in comparison with Ca(OH)_2_ paste.

The study involved two stages; (stage one): determination of the antibacterial potential of PS in liquid and gel phase against *E. faecalis* then compare it with Ca(OH)_2_ paste. This was followed by (stage two): assessment of MIC of the PS that showed the maximum ZOI.

The first stage of the study revealed that the PS cocktail mix is the most potent PS and was more effective than Ca(OH)_2_. The PS cocktail mix showed the largest ZOI both in solution and gel forms while stage two showed that the MIC of PS cocktail solution and gel were the same without any significant difference between them. Subsequently, the broth microdilution method was done on the PS solution only to confirm the MIC which was found to be similar.

The null hypothesis was rejected as the antibacterial effect of the *Lactobacilli* cocktail mix of the three supernatants (gel and solution) was significantly higher than the Ca(OH)_2_ paste.

*E. faecalis* was selected as an endodontic pathogen example due to its resistance to the high pH of Ca(OH)_2_ [6, 9]. In addition, it is one of the most frequently isolated bacteria from failed root canal treatments as it has a substantial role in persistent infection and is frequently encountered in primary infections [7, 47].

Calcium hydroxide Ultra Cal XS (Ca(OH)_2_) is the routinely used medication in root canal treatments, commercially available in the market, but has a limited ability against *E. faecalis* infection [13, 48]. Therefore, it was selected as a historical control for comparison with the new antibacterial agent.

Recently, probiotics had a growing role in the dental field owing to their antibacterial and anti-inflammatory potential in controlling infectious and inflammatory diseases like gingivitis and periodontitis [17, 27, 28, 49]. Three strains of *Lactobacilli* probiotics were selected *L. plantarum (LP), L. rhamnosus (LR) and L. acidophilus (LA)* as being the most commonly used probiotics in dental research [34, 38, 49].

Poloxamer 407 was chosen as a vehicle material for development of PS gel since it was used as an in-situ gel containing antibiotics in treatment of periodontal disease, also used in toothpastes, contraceptive gels and burn dressing materials [43–45]. The thermo-reversible properties of poloxamer gel 30% make it an appropriate drug delivery vehicle as it exists in gel form at room temperature and liquid form at low temperature mainly 4 °C [33, 43].

Although the study started with a concentration of 200 mg/ml PS in the first stage, 300 mg/ml was used as a higher concentration to compare the effectiveness of a concentration higher than 200 mg/ml, but it was found not significant. Therefore, this study recommends using another higher concentration like 400 mg/ml or more and comparing its efficiency with the 200 mg/ml concentration.

Agar well diffusion and broth microdilution are valid antimicrobial susceptibility testing methods that are commonly used to detect new antimicrobial agents [50, 51]. One of the drawbacks of the agar diffusion assay is that its result depends on the diffusibility of the tested agents [50], thus it is recommended to use another assessment method like the direct exposure test (DET) and compare its results with the agar diffusion test as Estrela et al. [52] performed in their study.

All PS supernatants showed a variable potential of growth inhibition on *E. faecalis* with the superiority of the PS cocktail mix indicating a possible synergistic effect of the three strains. These findings were supported by Plaza-Diaz [17], who reviewed the main mechanism of action of probiotics which included prevention of biofilm formation by competitive exclusion of pathogens and production of bacteriocin and other antimicrobial substances.

The results agreed with Seifelnasr’s study [32], which showed that *L.rhamnosus L. acidophilus, L. casei* had an inhibitory action on *E. faecalis* and are the most common used probiotics in commercial cocktail. Furthermore, Bohora et al. [33] who used commercial probiotics cocktails (Ecobion and Darolac), stated that probiotics effectively prevented the growth of *E. faecalis*. Another study held by the same author using individual probiotics strains (*L. rhamnosus* (ATCC 7469, *L. plantarum* (ATCC 8014),) and *Bifidobacterium bifidum* (ATCC 11,863)) showed the same result [34]. On the contrary, Hammad [31] used two strains of *Lactobacillus*, PTA 5289 and DSM 17,938 and found that probiotics had a non-significant effect on *E. faecalis*.

On comparing the (PS) cocktail mix (gel and solution) with 35%, Ultra Cal XS Ca(OH)_2_ paste against *E. faecalis*, Ca(OH)_2_ paste exhibited the least ZOI. The possible reason for this result could be due to the limited diffusion of Ca(OH)_2_ paste through blood agar because of its high viscosity, as the viscosity of the paste can affect the antibacterial activity [11].

However, Ultra Cal XS contains 3% propylene glycol and 2% methylcellulose [53, 54]. Propylene glycol is a viscous vehicle that reduces dispersion and diffusion due to its high molecular weight but maintains prolonged release and improves handling of the material [11, 55]. Methylcellulose is an aqueous stabilizing matrix that allows rapid dissociation of ions and improves the diffusibility and viscosity of the paste [11, 56].

Blanscet et al. [56] used the agar diffusion method to compare the antibacterial effect of Ca(OH)_2_ paste with different concentrations and vehicles on *E. faecalis* and found that the effect of 35% Ultra Cal XS Ca(OH)_2_ paste with aqueous methylcellulose was less than 60- 40% Ca(OH)_2_ but greater than 30% Vitapex (Neo-Dental Int, Federal Way, WA) with non-aqueous silicone vehicle.

The present study advocated that *Lactobacilli* PS could be used as an irrigant (liquid form) or an intracanal medication (gel form) as no significant differences were found between PS solution and gel. This study could be considered the baseline for using PS in endodontics as the minimum effective concentration of PS was determined, but further evaluations are needed in future studies.

Similar to the present study, El-Sayed et al. [35] and Kumari [36] et al. revealed that probiotics are potential natural irrigants. In future studies, it is recommended to compare the antibacterial efficacy of PS cocktail solution with the gold standard irrigant (sodium hypochlorite).

Moreover, Noushad et al. [37] reported that probiotics could be a promising intracanal medication that could inhibit *E. faecalis* more than and Ca(OH)_2_ but less than triple antibiotic paste.

Regarding limitations of the present study, the study was performed on *E. faecalis* in the Planktonic state; however, persistent bacteria are present in a biofilm form, which is harder to eradicate. Nevertheless, a tooth model is a more reliable method to assess antibacterial activity as intra-canal agents can be inactivated by dentin [57]. Another limitation, the culture and antimicrobial testing methods (agar diffusion assay and broth microdilution) are highly subjective. Results should have been confirmed by more accurate techniques as time kill assay.

Benbelaïd et al. [47] evaluated the antimicrobial effect of essential oil against *E. faecalis* in both planktonic and biofilm states and determined biofilm eradication concentration (BEC). Accordingly, it is recommended to assess the BEC of the PS cocktail against *E. faecalis* and compare it with the MIC.

Future studies can evaluate PS cocktail gel with different vehicles to detect the ideal delivery vehicle of PS for clinical application. This study was the first to use a cocktail mix of three supernatants of PS in gel form and assess its antibacterial activity, besides determining the MIC of the PS against *E. faecalis.*

## Conclusion

*Lactobacilli* PS cocktail revealed a significant antibacterial effect against *E. faecalis*, suggesting them as a promising antibacterial agent that can be used as an irrigant or an intracanal medication by using poloxamer 407 as a vehicle.

## Data Availability

The raw data of the present study is available at: http://dx.doi.org/10.6084/m9.figshare.18858008

## Abbreviations

*E*. *faecalis*: *Enterococcus* *faecalis*
Ca(OH)_2_: Calcium hydroxide
WHO: The World Health Organization
GRAS: Generally recognized as safe
PRILE: Preferred reporting items for laboratory studies in endodontology
MRS: DeMan rosa sharpe
CFS: Cell-free supernatant
PS: Probiotics supernatants
LP: *Lactobacillus* *plantarum*
LR: *Lactobacillus* *rhamnosus*
LA: *Lactobacillus* *acidophilus*
ZOI: Zone of inhibition
MIC: Minimum inhibitory concentration
BMD: Broth microdilution
CLSI: Clinical Laboratory Standards Institute
CAMHB: Cation-adjusted Mueller–Hinton broth
